# Postoperative Delirium after Reconstructive Surgery in the Head and Neck Region

**DOI:** 10.3390/jcm11226630

**Published:** 2022-11-09

**Authors:** Juergen Taxis, Steffen Spoerl, Andreas Broszio, Jonas Eichberger, Elisabeth Grau, Johannes Schuderer, Nils Ludwig, Maximilian Gottsauner, Gerrit Spanier, Annika Bundscherer, Torsten E. Reichert, Tobias Ettl

**Affiliations:** 1 Department of Cranio- and Maxillofacial Surgery, Hospital of the University of Regensburg, Franz-Josef-Strauß-Allee 11, 93053 Regensburg, Germany; 2 Department of Anesthesiology, Hospital of the University of Regensburg, Franz-Josef-Strauß-Allee 11, 93053 Regensburg, Germany; 3 Department of Oral and Maxillofacial Surgery, Leipzig University Medical Center, Liebigstraße 12, 04103 Leipzig, Germany

**Keywords:** delirium, head neck squamous cell carcinoma, HNSCC, free flap, Charlson Comorbidity Index

## Abstract

Postoperative delirium (POD) is an acute and serious complication following extended surgery. The aim of this study was to identify possible risk factors and scores associated with POD in patients undergoing reconstructive head and neck surgery. A collective of 225 patients was retrospectively evaluated after receiving reconstructive surgery in the head and neck region, between 2013 to 2018. The incidence of POD was examined with regards to distinct patient-specific clinical as well as perioperative parameters. Uni- and multivariate statistics were performed for data analysis. POD occurred in 49 patients (21.8%) and was strongly associated with an increased age-adjusted Charlson Comorbidity Index (ACCI) and a prolonged stay in the ICU (*p* = 0.009 and *p* = 0.000, respectively). Analogous, binary logistic regression analysis revealed time in the ICU (*p* < 0.001), an increased ACCI (*p* = 0.022) and a Nutritional Risk Screening (NRS) score ≠ 0 (*p* = 0.005) as significant predictors for a diagnosis of POD. In contrast, the extent of reconstructive surgery in terms of parameters such as type of transplant or duration of surgery did not correlate with the occurrence of POD. The extension of reconstructive interventions in the head and neck region is not decisive for the development of postoperative delirium, whereas patient-specific parameters such as age and comorbidities, as well as nutritional parameters, represent predictors of POD occurrence.

## 1. Introduction

Postoperative delirium (POD) is an acute, potentially life-threatening diagnosis with cerebral dysfunction, characterized by alternating sequences of disordered thinking, altered stages of consciousness, and varying degrees of inattention [1,2]. Three different forms have been scientifically described: a hyperactive (agitation, aggressiveness, and hallucination); hypoactive (reduced attention, lethargy, and apathy); as well as a mixed form of both types [3,4,5]. The development of POD is a well-known complication that occurs after major surgery and prolonged anesthesia during an intensive care unit (ICU) stay [6]. Hereby, current literature describes the incidence of POD after head and neck surgery to range between 11% and 26% [7]. Related to that, the total amount of patients who received major surgery was described to postoperatively develop delirium in 10–92% of cases [8]. POD not only causes higher costs for the healthcare system, it is also closely associated with a higher number of complications and a prolonged stay in hospital [9]. Furthermore, the occurrence of POD is linked to a higher mortality rate compared to patients without delirium [10,11]. In this regard, the key to reducing the risk and severity of POD is the early identification of patients at a high risk of POD through active perioperative management and the postoperative recognition of delirium in the ICU [12,13,14]. However, delirium is still often misdiagnosed or not diagnosed at all [15].

Patients who have undergone free flap reconstructive surgery after major tumor resection in the head and neck area have a higher risk of acquiring delirium due to the surgery duration, the increased rate of malnutrition, and alcohol abuse [7,16]. While many studies have focused primarily on the complications surrounding flap surgery, relatively few have addressed medical complications such as POD in this patient population. Preliminary work on these defined factors, such as older age, male gender, tobacco consumption, the duration of surgery, blood transfusions, the type of graft, and neck dissections, could be described as potential risk factors for POD after extensive tumor surgery [6,7,17,18,19,20].

Therefore, the aim of this study was to analyze the occurrence of POD, including further factors and scores, in patients who have undergone reconstructive surgery using a microvascular or distant graft in the head and neck area.

## 2. Materials and Methods

### 2.1. Patient Selection and Data Collection

**Figure A1:**
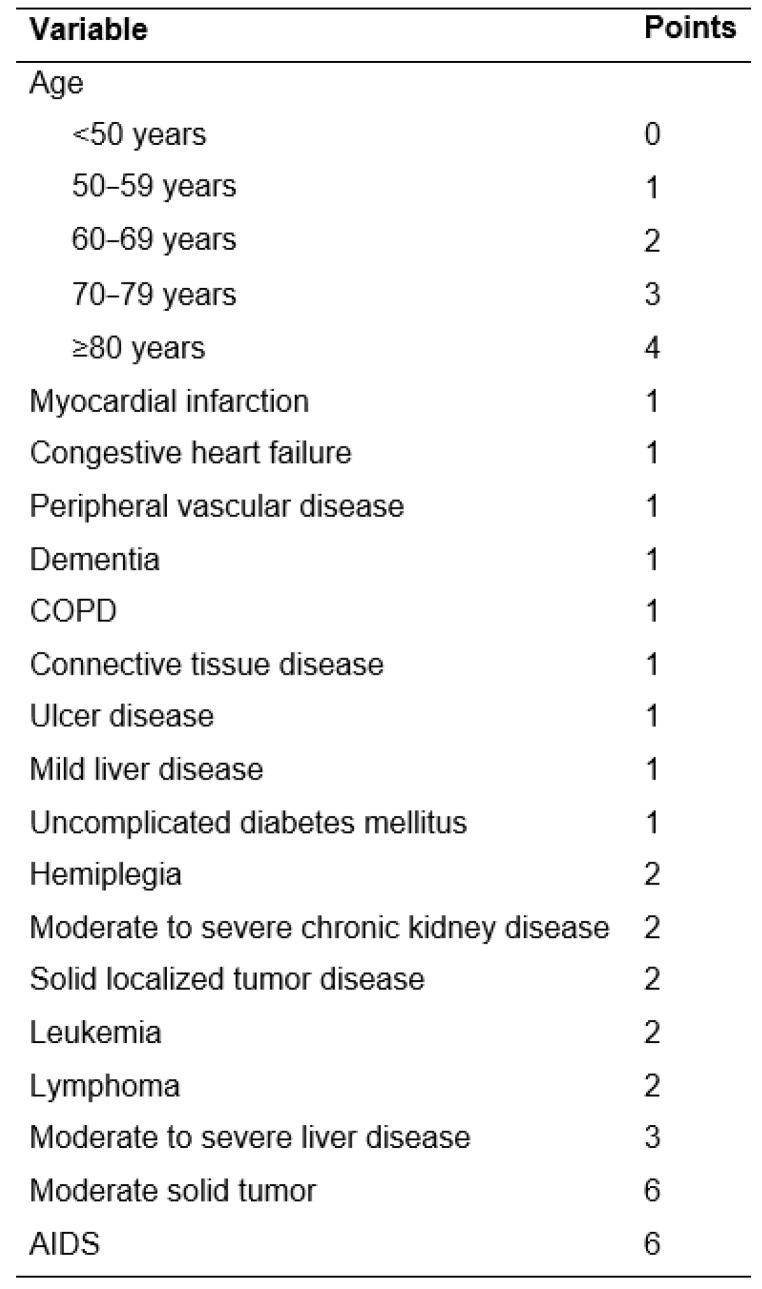
Charlson Comorbidity index; incorporated diseases, weighting range from 1 to 6 points. To adjust for age, every decade after 40 years is reflected by 1 point (maximum weight for age: 4 points).

This retrospective monocentric study was conducted at the Department of Cranio- and Maxillofacial Surgery, University Hospital Regensburg, Germany. A total of 494 patients who were reconstructed using free and distant flaps in the oral cavity and head and neck region, between 2013 and 2018, were evaluated. From this collective, 225 patients had a complete delirium documentation as well as a comprehensive survey of other factors mentioned below and were used for the final examination. Pre-, peri- and postoperative data as possible risk factors for POD were collected for the analysis, and included: the degree of comorbidity; nicotine and alcohol abuse; diagnosis for surgical intervention; the region of surgery; previous surgeries in the head and neck; the type and size of reconstruction; distinct flap type; flap success; duration of surgery; tracheostomy passed; wound healing disorders; duration of ICU stay; and Nutritional Risk Screening (NRS) score after sedation. The degree of comorbidity was evaluated using the age-adjusted Charlson Comorbidity Index (ACCI) as previously described in the literature and patients were classified according to the score (Figure A1) [21]. A successful graft was defined as a functioning graft with no signs of graft loss up to 6 weeks after surgery. The diagnosis of POD, represented by a disturbance of attention, awareness, and cognition, that developed over a short period of time and whose severity fluctuated over the course of a day, was made by both an experienced nurse and the attending physician [2]. The diagnosis was determined in the ICU on the basis of the RASS score and the NRS score. Patients diagnosed with alcohol withdrawal delirium were not included in the study.

### 2.2. Statistical Analysis

Statistical data were collected using IBM SPSS Statistics 26 (IBM Corp. SPSS for Mac, Armonk, NY, USA). Univariate analysis was performed using chi-square test to compare different groups of outcome parameters in the presence or absence of certain pre- and perioperative risk factors. Uni- as well as multivariate correlations/regression analysis between the occurrence of delirium and the individual variables were carried out. Multivariate regression analysis was based on the binary logistic regression method. The significance level was considered as *p* < 0.05.

## 3. Results

### 3.1. Clinicopathological Characteristics of the Patient Cohort

**Figure 1:**
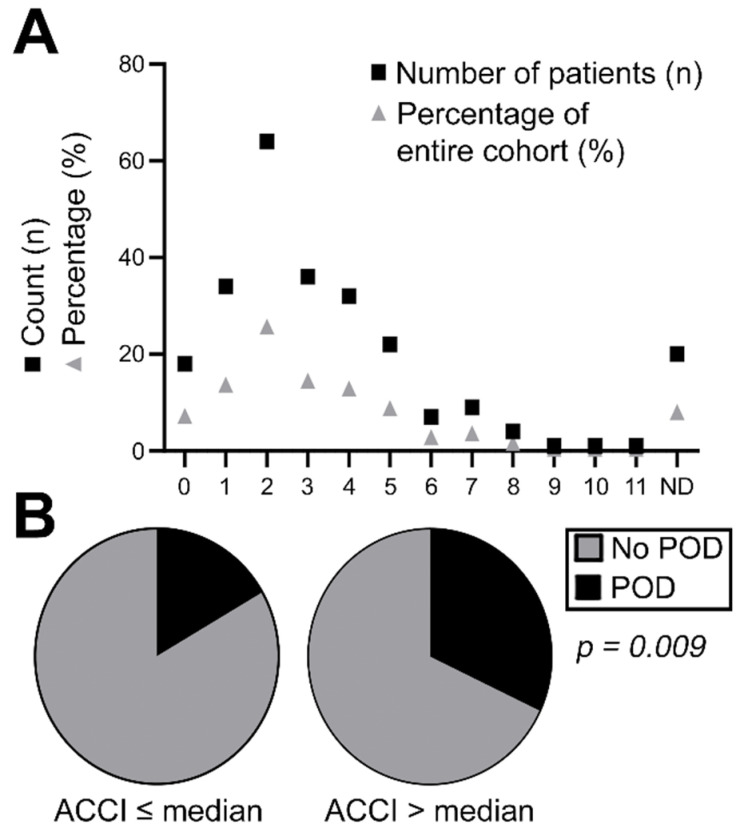
(**A**) Distribution of age-adjusted Charlson Comorbidity index with a mean of 3.0 points and a range between 0 and 11 points, (**B**) impact of age-adjusted Charlson Comorbidity index on incidence of POD. ND = not determinable.

The present retrospective cohort study on patients having received pedicled or microvascular flap transfer in the head and neck region comprises 225 cases. Table 1 shows patient characteristics of the entire cohort. In all, 77 patients were female (34.2%), whereas the majority of the cohort was comprised by male patients (65.8%). POD was diagnosed in 49 patients (21.8%). A total of 99 patients (44.0%) had a positive history of nicotine and alcohol abuse, the mean age was 62.4 years (range: 20–89 years). The most common diagnosis for surgical intervention was oral squamous cell carcinoma (OSCC; 84.4%) followed by osteoradionecrosis (ORN; 10.7%). The majority of patients received reconstructive surgery based on microvascular flap transfer (88.0%), whereas pedicled transplants such as the pectoralis major myocutaneous flap was applied in 10.8% of cases. Additionally, the ACCI was retrospectively calculated for each patient. Hereby, the mean ACCI was 3 points with a range between 0 and 11 points (Figure 1A).

### 3.2. Association of Clinicopathologic Characteristics with Diagnosis of POD

**Figure 2:**
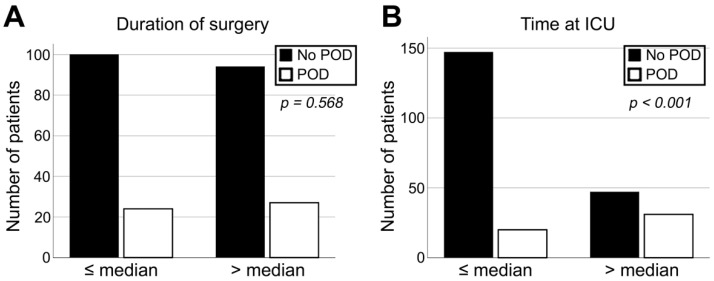
Impact of duration of surgery (**A**) as well as time at intensive care unit (ICU) (**B**) on occurrence of POD.

Univariate correlation analysis revealed a highly significant correlation between the occurrence of POD and elevated ACCI in our retrospective cohort (*p* = 0.009). The diagnosis of POD significantly prolonged patients’ stay in the ICU (Table 1, Figure 2B). Furthermore, a trend to develop POD was observed for patients with a positive history of nicotine and alcohol abuse (*p* = 0.093) and a pathologic Nutritional Risk Screening (NRS) score (*p* = 0.052; Table 1).

### 3.3. Correlation of Flap-Related Parameters with Diagnosis of POD

**Figure 3:**
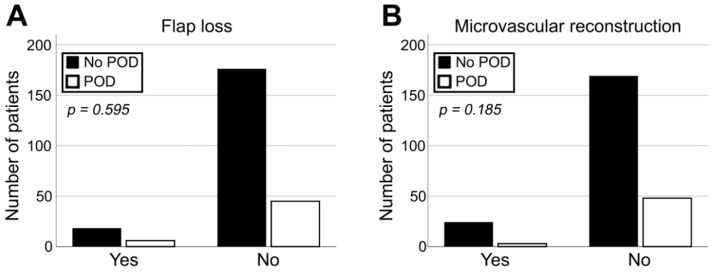
Impact of diagnosis of POD on occurrence of flap loss (**A**) and association between type of reconstruction and incidence of POD (**B**).

As a major aspect of the present cohort study, flap-related parameters were evaluated with regards to the development of POD. Hereby, pedicled and microvascular transplants showed no significant difference in terms of correlation with POD (*p* = 0.185; Table 1, Figure 3B). Chi-square-test showed no significant correlation for duration of flap surgery, the size of reconstruction, and a performed tracheostomy in univariate statistics (*p* = 0.568, *p* = 0.642, and *p* = 0.660, respectively; Figure 2A, Table 1). Furthermore, the development of POD did not correlate with flap loss or impaired wound healing after reconstructive surgery in the head and neck area (*p* = 0.568 and *p* = 0.642, respectively; Table 1, Figure 3A).

### 3.4. Binary Logistic Regression Analysis of POD Diagnosis and Clinicopathological Parameters

In addition to univariate statistics, binary logistic regression was applied to analyse the occurrence of POD with regards to different clinicopathological parameters. Hereby, in line with previous results, time in the ICU was highly significantly correlating with the diagnosis of POD after reconstructive surgery (*p* < 0.001; Table 2). Additionally, an elevated ACCI, as well as a NRS score ≠ 0 correlated significantly with POD (*p* = 0.022 and *p* = 0.005, respectively; Table 2). While patients with nicotine and alcohol abuse were more likely to develop POD, no significant correlation was observed for flap loss, impaired wound healing, or the type of flap transfer (Table 2).

## 4. Discussion

The incidence of POD in our study was 21.8% and had a similar diagnostic rate as previously reported in the literature [17,20]. Any delirium that occurred was treated symptomatically according to the current guidelines of the German Society for Anaesthesia and Intensive Care Medicine [22]. At the beginning, patients were supported by orientation and supportive measures, such as maintaining a day-night rhythm. If sedation or analgesia was required, the selective α2-agonist dexmedetomidine was used, as patients showed a significantly shorter duration of delirium in previous studies [23,24]. For the attenuation of autonomic sympathetic hyperactivity, α-blockers such as clonidine were used. In the case of productive psychotic symptoms, haloperidol, risperidone, and olanzapine were applied in low doses.

A prolonged postoperative stay in the ICU was linked with a higher incidence rate of POD and is in agreement with the results of Kolk et al. [19]. Likewise, a high Charlson score and thus a higher comorbidity as well as older age had a positive influence on POD. In contrast to Yamagata et al., Shah et al., Zhu et al., and Densky et al., and in agreement with Booka et al., we were not able to demonstrate a higher incidence of POD after prolonged surgical procedure [6,7,16,17,20]. In this regard, even extended free flap reconstructive surgery, which are not unusual to last longer than 6 h, did predominantly not result in patients showing signs of POD. Therefore, the choice of graft cannot be considered as a risk factor for POD in our patient cohort, which is a contradictory finding in comparison to previous studies [19]. In detail, we could not detect any difference in patients’ frequency to develop a POD between microvascular flaps and pedicled alternatives such as the pectoralis flap.

A pathological NRS score showed up in our results with an increased incidence of POD. Although malnutrition cannot be easily remedied shortly before the usually urgent surgery, it represents an opportunity for improvement in care, provided that the patient remains an inpatient for a certain period of time pre-operatively.

The ACCI, cited over 8800 times in the literature, represents an extensively validated model for the assessment of comorbidities [25,26]. Especially for tumors in the head and neck region, the ACCI has proven to be a valid prognostic indicator to predict the outcome of cancer patients [27]. However, some modifications may be necessary for applicability in head and neck surgery. For example, AIDS as a heavily weighted parameter of comorbidity is not necessarily up-to-date any more, since it can no longer be compared with distant metastatic tumor disease in terms of mortality and thus in its severity—at least in developed countries [28]. Furthermore, there are other scores for determining comorbidity such as the Elixhauser comorbidity score or the comorbidity-polypharmacy score [29,30]. Besides, of all criticism, the ACCI potently predicted the occurrence of POD after major reconstructive surgery in the head and neck area and thereby is, at least in our view, a valuable and easily accessible tool to assess comorbidity and screen for higher patients’ risk to develop POD.

Of course, one must bear in mind that delirium diagnosis is partly subjective in nature and depends on the experience of the nurse or the attending physician. Although 494 operated patients were initially considered in the observed period, only 225 patients remained, since the majority of patients did not have sufficient delirium documentation, which therefore leads to the fact that POD could not be 100 per cent confirmed or excluded. Accordingly, the retrospective conception represents the greatest weakness of this study, since it was necessary to rely absolutely on the electronical and paper-based documentation of the cases and questionable entries thereby could not be included in the study. Furthermore, there was no individual risk assessment of delirium in the patients preoperatively, which could certainly show an early hint for clinicians to be aware of an increased patient’s risk to develop POD. In addition, there was a profound history of noxious substance abuse in our patient collective, which may well occur more frequently with the primary diagnoses treated here. However, this was not infrequently incorrect due to possible misreporting of the amount of nicotine and alcohol consumption and, moreover, need not correspond to the normal population. Compared with the delirium cohorts from other surgical specialties, however, the incidence was similar [31,32].

## 5. Conclusions

The degree of morbidity of each individual patient should be considered with greater caution as a major predictor for developing POD after reconstructive flap surgery in the head and neck region. In contrast, as a fundamental result of the present retrospective cohort study, the sole and especially the extent of flap surgery in the head and neck do not inevitably correlate with the occurrence of POD. This should be kept in mind for planning extensive reconstructive interventions in the head and neck region.

## Figures and Tables

**Table 1:** Clinicopathological characteristics of the patient cohort ^a^.

		Clinical Diagnosis of Postoperative Delirium						
		No		Yes		Total		χ^2^
		N	%	N	%	N	%	*ρ*
Sex	Female	61	34.7%	16	32.7%	77	34.2%	0.756
	Male	115	65.3%	33	67.3%	148	65.8%	
ACCI	≤Median	125	71.0%	25	51.0%	150	66.7%	0.009
	>Median	51	29.0%	24	49.0%	75	33.3%	
Positive history of nicotine and alcohol abuse	No	103	58.5%	23	47.0%	126	56.0%	0.093
	Yes	73	41.5%	26	53.0%	99	44.0%	
Diagnosis for surgery	OSCC	147	83.5%	43	87.8%	190	84.4%	
	ORN	20	11.4%	4	8.2%	24	10.7%	
	MRONJ	2	1.1%	1	2.0%	3	1.3%	
	Osteomyelitis (not ORN or MRONJ)	6	3.4%	1	2.0%	7	3.1%	
	Other	1	0.6%	0	0.0%	1	0.4%	
Site of reconstruction	Mandible	57	32.6%	17	35.4%	74	33.0%	
	Upper alveolus and gingiva & Hard palate	20	11.4%	5	10.4%	25	11.2%	
	Tongue & Floor of mouth	64	36.6%	20	41.7%	84	37.5%	
	Face & Neck	15	8.6%	2	4.2%	17	7.6%	
	Buccal mucosa	19	10.6%	4	8.3%	23	10.2%	
Previous head and neck surgery	No	93	52.8%	29	59.2%	122	54.2%	0.239
	Yes	83	47.2%	20	40.8%	103	45.8%	
Microvascular surgery	No	21	12.0%	3	6.1%	24	10.7%	0.185
	Yes	154	88.0%	46	93.9%	200	89.3%	
Flap type	Radial forearm flap	75	42.6%	28	57.1%	103	45.8%	
	Anterolateral thigh flap	18	10.2%	3	6.1%	21	9.3%	
	Free upper arm flap	1	0.6%	0	0.0%	1	0.4%	
	Latissimus dorsi flap	7	4.0%	2	4.1%	9	4.0%	
	Free fibula flap	47	26.7%	11	22.4%	58	25.8%	
	Deep circumflex iliac artery flap	4	2.3%	2	4.1%	6	2.7%	
	Scapular flap	1	0.6%	0	0.0%	1	0.4%	
	Pectoralis major myocutaneos flap	19	10.8%	3	6.1%	22	9.8%	
	Submental island flap	4	2.3%	0	0.0%	4	1.8%	
Size of reconstruction	≤Median	74	49.7%	21	53.8%	95	50.5%	0.642
	>Median	75	50.3%	18	46.2%	93	49.5%	
Flap success	No	18	9.3%	6	11.8%	24	9.8%	0.743
	Yes	176	90.7%	45	88.2%	221	90.2%	
Impaired wound healing	No	145	74.7%	32	62.7%	177	72.2%	0.089
	Yes	49	23.3%	19	37.3%	68	27.8%	
Tracheostomy	No	96	49.5%	27	52.9%	123	50.2%	0.660
	Yes	98	50.5%	24	47.1%	122	49.8%	
Time at ICU	≤Median	147	75.8%	20	39.2%	167	68.2%	0.000
	>Median	47	24.2%	31	60.8%	78	31.8%	
Duration of surgery	≤Median	100	51.5%	24	47.0%	124	50.6%	0.568
	>Median	94	48.5%	27	53.0%	121	49.4%	
NRS score after end of sedation	0	141	80.6%	29	67.4%	170	78.0%	0.052
	≠0	34	19.4%	14	32.6%	48	22.0%	

^a^ ACCI = Age-adjusted Charlson Comorbidity Index, OSCC = Oral Squamous Cell Carcinoma, ORN = Osteoradionecrosis, MRONJ = Medication-Related Osteonecrosis, ICU = Intensive Care Unit.

**Table 2:** Binary logistic regression of different clinicopathological parameters related to clinical diagnosis of delirium ^a^.

Factor	*p* Value	Odds Ratio	95% Confidence Interval, Lowest Value	95% Confidence Interval, Highest Value
Duration of surgery	0.392	1.467	0.610	3.529
ACCI	0.022	2.579	1.144	5.816
Sex	0.812	0.898	0.369	2.183
Time at ICU	<0.001	4.753	2.172	10.403
Impaired wound healing	0.390	1.489	0.601	3.690
Positive history of nicotine and alcohol abuse	0.068	2.248	0.941	5.368
Microvascular surgery	0.187	2.700	0.618	11.796
Previous head and neck surgery	0.846	0.913	0.366	2.280
Flap success	0.801	1.183	0.320	4.381
Tracheostomy	0.391	0.666	0.264	1.685
Postoperative NRS score	0.005	3.678	1.496	9.043

Model summary: x^2^ = 34.470, *p* < 0.001, Nagelkerke’s R^2^ = 0.239; ^a^ ACCI = Age-adjusted Charlson Comorbidity Index, ICU = Intensive Care Unit, NRS = Nutritional Risk Screening.

## Data Availability

Data can be obtained by scientists that conducted the work independently from the industry, on request. Data are not stored on publicly available servers.
